# A Lightweight Convolutional Neural Network Architecture Applied for Bone Metastasis Classification in Nuclear Medicine: A Case Study on Prostate Cancer Patients

**DOI:** 10.3390/healthcare8040493

**Published:** 2020-11-18

**Authors:** Charis Ntakolia, Dimitrios E. Diamantis, Nikolaos Papandrianos, Serafeim Moustakidis, Elpiniki I. Papageorgiou

**Affiliations:** 1 Department of Computer Science and Biomedical Informatics, School of Science, University of Thessaly, 35100 Lamia, Greece; didiamantis@uth.gr; 2 Department of Energy Systems, Faculty of Technology, Geopolis Campus, University of Thessaly, Larissa-Trikala Ring Road, 41500 Larissa, Greece; elpinikipapageorgiou@uth.gr; 3 AIDEAS OÜ, Narva mnt 5, 10117 Tallinin, Estonia; s.moustakidis@aideas.eu

**Keywords:** machine learning, convolutional neural network, bone metastasis classification, deep learning, medical image, nuclear medicine, lightweight look-behind fully convolutional neural network

## Abstract

Bone metastasis is among the most frequent in diseases to patients suffering from metastatic cancer, such as breast or prostate cancer. A popular diagnostic method is bone scintigraphy where the whole body of the patient is scanned. However, hot spots that are presented in the scanned image can be misleading, making the accurate and reliable diagnosis of bone metastasis a challenge. Artificial intelligence can play a crucial role as a decision support tool to alleviate the burden of generating manual annotations on images and therefore prevent oversights by medical experts. So far, several state-of-the-art convolutional neural networks (CNN) have been employed to address bone metastasis diagnosis as a binary or multiclass classification problem achieving adequate accuracy (higher than 90%). However, due to their increased complexity (number of layers and free parameters), these networks are severely dependent on the number of available training images that are typically limited within the medical domain. Our study was dedicated to the use of a new deep learning architecture that overcomes the computational burden by using a convolutional neural network with a significantly lower number of floating-point operations (FLOPs) and free parameters. The proposed lightweight look-behind fully convolutional neural network was implemented and compared with several well-known powerful CNNs, such as ResNet50, VGG16, Inception V3, Xception, and MobileNet on an imaging dataset of moderate size (778 images from male subjects with prostate cancer). The results prove the superiority of the proposed methodology over the current state-of-the-art on identifying bone metastasis. The proposed methodology demonstrates a unique potential to revolutionize image-based diagnostics enabling new possibilities for enhanced cancer metastasis monitoring and treatment.

## 1. Introduction

Bones, along with lung and liver, are identified as the most common sites for cancer metastasis, causing morbidity especially to patients with advanced-stage cancer. Early diagnosis permits accurate patient management and treatment decision making that consecutively can lead to improvement of patient’s condition and quality of life and the rise of their survival rates [1,2].

When it comes to bone metastasis (BS) diagnosis, various techniques are available nowadays such as magnetic resonance imaging (MRI), radiography, and computed tomography (CT) [3]. However, whole-body bone scans (WBS) have been identified as the standard method for the detection of bone metastatic tumors due to lower costs and equivalent performance compared to the other aforementioned techniques [4]. The so-called hot spots, which are areas receiving higher intensity signals than the surroundings, signify the potential abnormalities in WBS images and contribute to the diagnosis of BS. Hence, the accurate and effective WBS interception for BS diagnosis remains a challenging and subjective task that demands experience since hot spots can be presented even to patients that do not suffer from BS [5]. To this end, a methodology that will be able to identify hot spots relevant to BS is of paramount importance for supporting the treatment decision making.

Over the last decade, artificial intelligence (AI) has been applied in diagnostic disciplines to support medical decision-making and to interpret medical data, such as medical images [6,7,8]. Computer-aided detection of BS was proposed for bone scintigraphy images based on a parallelepiped classification method to map the radionuclide distribution in [9]. Another automated diagnosis for BS was proposed in [10] exploiting multi-view bone scans with attention-augmented deep neural networks. The model contains three parts that aim to extract, aggregate, and classify high-level features in a data-driven manner. An automated diagnostic tool, ‘CADBOSS’, was proposed in [11] for BS using WBS images. The system was implemented with an Artificial Neural Network (ANN) classifier to identify potential metastases in cancer patients. A segmentation method for predicting the contour and location of the lesion areas of BS in CT images has been also developed in [12]. The study modifies the holistically nested edge detection (HED) network to match the characteristics of BS. Diagnosis of breast cancer metastasis in bones via deep learning was presented in [13] taking advantage of WBS. The study compared various convolutional neural networks (CNN) models for solving the binary classification problem of bone metastasis from breast. Another approach was proposed for BS diagnosis by applying and compared fast convolutional neural networks trained on bone scintigraphy images [14]. Extending this study, a further investigation was performed for simpler, faster, and more accurate CNN architectures based on CNN hyper-parameter selection and fine-tuning [15]. In another study [16] the skeletal-related events were investigated on cancer patients with BS using linear regression (LR), decision trees (DT), and support vector machines (SVM).

Recently, due to the technological advancements in medical imaging, positron emission tomography (PET) has been also recognized as an efficient method of detecting cancer cells. This method combined with computed tomography (CT) could provide images of high-resolution [17]. Focusing on PET/CT imaging, a deep convolutional neural network has been implemented, based on VGG19 architecture, for automatically differentiating benign and malignant lesions in sodium fluoride positron emission tomography (^18^F-NaF PET/CT) images of patients with metastatic prostate cancer [18]. Another CNN-based system was proposed to detect malignant findings in FDG PET-CT examinations in a retrospective study including 3485 sequential patients. A neural network model similar to ResNet24 was also employed to solve the three-classes classification problem consisting of the categories: (i) benign, (ii) malignant, and (iii) equivocal in [19]. Apart from CNN approaches, random forest (RF) was adopted for addressing the BS classification problem in combination with a threshold-based method for detection [20]. The proposed methodology was developed in the context of an automatic evaluation of ^18^F-NaF PET/CT scans for bone metastases in patients with prostate cancer.

Despite the significant number of studies focusing on AI adoption to medicine, there are still limitations of the current AI techniques that need to be overcome. In the field of computer vision (CV) convolutional neural networks (CNNs), such as VGG-16 [21] and ResNet [22], can extract features that better represent the input space when compared to conventional hand-crafted features [23]. While the classification performance of deep CNNs is remarkable, the training procedure requests a large amount of training data to be available. This is directly derived from the large number of free parameters that are required to be trained. While in many cases, such as natural images, this is viable, data availability in the medical domain is limited mainly due to ethical concerns. Another drawback of a large number of free parameters is that they are directly connected to the computational performance of the trained model, which limits their use on high-end computational platforms typically equipped with graphics processing units (GPUs). To this end, to enable the use of CNNs on mobile and embedded devices, there has been work towards the minimization of the number of free parameters and thus towards the increase of computational performance and decrease of their memory footprint. Examples include MobileNetV2 [24], which achieves a trade-off between computational performance and classification accuracy.

Recently, in the domain of gastrointestinal tract abnormality detection look-behind fully convolutional neural network (LB-FCN) has achieved state-of-the-art results. The network is characterized by multi-scale feature extraction modules composed of parallel convolutional layers and residuals connections across the network. This enables the network to learn features under different scales increasing the overall generalization performance [25]. In our study, we employed the lightweight look-behind fully convolutional neural network (LB-FCN *light*) architecture, which is a revised version of the original LB-FCN, focused on the computational performance reduction by decreasing the number of required free parameters. The lightweight version of LB-FCN has been already used for mobile applications such as staircase detection in natural images [13]. Variation of multiscale feature extraction and the low number of free parameters enables the network to generalize well, even when the number of training samples is limited. In this paper, the proposed LB-FCN *light* network was evaluated and compared with state-of-the-art pre-trained CNN networks of the recent literature for addressing the classification problem of patients with prostate cancer (P-Ca) for an assisted BS diagnosis. The main contributions of this study by adopting the use of a lightweight CNN is in the followings:

- Decrease the number of free parameters.
- Achieve high classification accuracy with small datasets.
- Decrease the training time needed for convergence.
- Decrease the complexity of the network thus enabling its mobile application.
- Establish a future research direction that will extend the applicability of the method to other types of scintigraphy.

The rest of this study is structured as follows: In Section 2 the dataset used in the research and the proposed methodology are presented. Section 3 presents the results achieved, while conclusions and future work are provided in Section 4.

## 2. Materials and Methods

### 2.1. Dataset of Whole-Body Scan Images

This research study contains retrospective patient records whose development is in accordance with the Declaration of Helsinki. The study was approved by the Board Committee Director of the Diagnostic Medical Center “Diagnostiko-Iatriki A.E.” Dr. Vassilios Parafestas and the requirement to obtain informed consent was waived by the Director of the Diagnostic Center due to its retrospective nature. Nuclear medicine physician and co-author of this paper, Dr. Nikolaos Papandrianos, who has 15 years of experience in bone scan interpretation, was mainly involved and contributed to the dataset collection and pre-processing, differential diagnosis for whole-body scans interpretation, and patient group characterization.

In this study, 817 male patients with prostate cancer (P-Ca) participated and examined with whole-body scintigraphy images for bone cancer metastasis. In total, 908 images were selected in the Nuclear Medicine Department of the Diagnostic Medical Center ‘Diagnistiko-Iatriki A.E.’ in Larissa, Greece from June 2013 until June 2018. The patient scanning was performed with a Siemens gamma camera Symbia S series SPECT System (Siemens, Enlargen, Germany) with two heads with low energy high-resolution (LEHR) collimators and with Syngo VE32B software (Siemens Healthcare, Forchheim, Germany). Anterior and posterior digital views of 1024×256 pixels resolution were captured by using a whole-body field.

A data pre-processing step of the scanned images was considered necessary due to the existence of artifacts and non-related to bone findings, such as medical accessories, radioisotope drugs, or urine accumulation [26,27]. The pre-processed dataset includes images from P-Ca patients with and without bone metastasis, or other benign etiologies as to the diagnosis, such as degenerative joint disease, benign fractures, and inflammation [28]. The degenerative changes are present in the whole body scans because 99mTc-MDP accumulates in response not only to the tumor but also to the reported benign findings [29].

The procedure followed by the experienced nuclear medicine physician to categorize patients into three classes/groups (as malignant (bone metastasis), degenerative changes, and normal) was as follows: Initially, by inspecting the provided image dataset, the NC physician was able to determine the normal bone scans which were characterized from metastasis absence. Next, following the typical scintigraphic patterns for bone metastasis (i.e., solitary focal lesions and multiple local lesions), as reported in [30], the nuclear medicine physician easily recognized certain features on the provided scintigraphy images, which helped him to define these image scans as malignant. Thus, he differentiated them from those that are characterized in the relevant literature as equivocal [31].

In the case of the equivocal group of image scans, further investigation using localized radiological examination such as computed tomography (CT) or magnetic resonance imaging (MRI) was requested by the physician to distinguish benign-degenerative (fracture, Paget’s, degenerative joint disease, etc.) from malignant (metastatic) origin patients [32]. The group of patients diagnosed with degenerative changes such as degenerative joint diseases (which include knee, hand, wrist, shoulder, and bones of the feet), or degenerative changes in the spine, formed the category “degenerative” (benign). The rest of the cases characterized as malignant from the aforementioned radiological examination were added to the previously defined “malignant” category/group.

**Figure 1:**
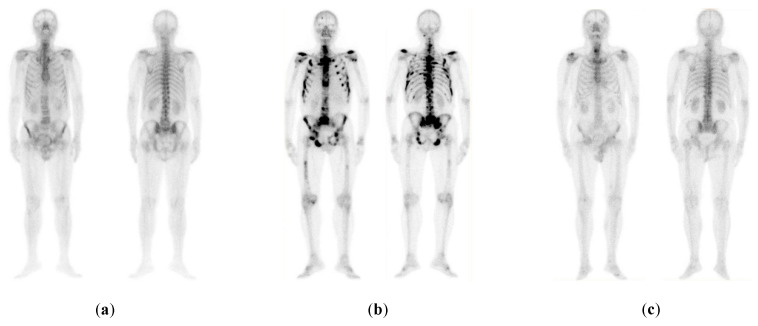
Examples of the three categories of prostate cancer (P-Ca) patients: (**a**) normal (metastasis absent); (**b**) malignant (metastasis present); (**c**) benign-degenerative (no metastasis, but image includes degenerative lesions/changes) [14].

Hence, these three classes were adopted in this study. In this study, 778 images were chosen where 328 illustrate bone metastasis, 271 degenerative alterations, and 179 without any bone metastasis findings (normal). Figure 1 illustrates representative cases of each one of the three categories.

### 2.2. The Proposed Methodology

**Figure 2:**
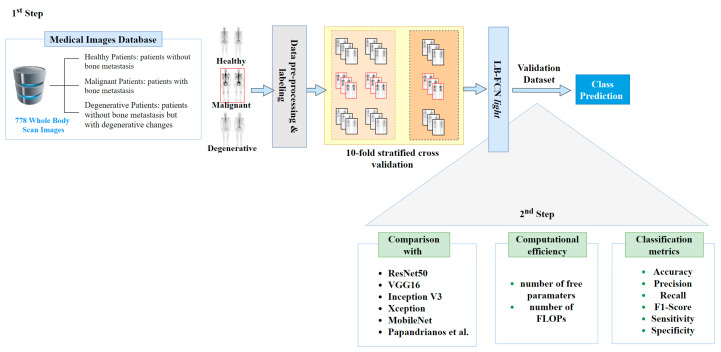
Methodology pipeline.

This study was divided into two steps (Figure 2): (i) the training and validation of the LB-FCN *light* model; and (ii) its comparison with state-of-the-art CNN architectures. In the first step, a pre-processing step was performed for data curation. In this process, the Red-Green-Blue (RGB) images were transformed to grayscale and a nuclear medicine doctor labeled the data based on three pre-determined classes, namely malignant, degenerative, and healthy. Then, data were normalized to achieve a scalable dataset in which the proposed LB-FCN *light* was trained.

**Figure 3:**
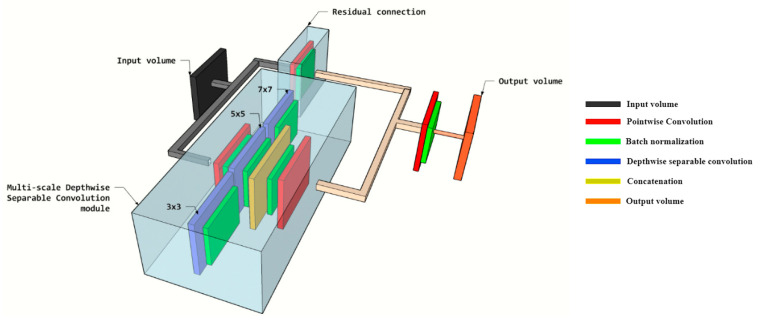
3D representation of the main multi-scale building block of look-behind fully convolutional neural network (LB-FCN) light architecture adopted in this study.

The design architecture of the adopted LB-FCN *light* is based on the initial LB-FCN [25] while a lightweight version of the LB-FCN was adopted [33] to decrease the architecture complexity, the number of free parameters and required floating-point operations (FLOPs). LB-FCN *light* was compared to conventional and pre-trained CNN architectures [34,35] used to solve the classification problem of bone metastasis from P-Ca patients [14]. Light LB-FCN follows the FCN network design [21] and it is based on the presence of depth wise separable convolutions among the convolutional layers of the network. In contrast with the conventional convolution where the filters are connected on the entire depth of the input channels, the filter in the depth wise convolution is applied on each channel separately followed by a 1×1 pointwise convolution for connecting the filters. The LB-FCN *light* that was used in our study is composed of four multi-scale blocks and three residual connections (Figure 3). In total the network is composed of 0.3 × 10^6^ free parameters and requires 0.6 × 10^6^ FLOPs for an inference.

In the second step, a comparison among the LB-FCN *light* and state-of-the-art CNN models, such as RESNET50, VGG16, Inception V3, Xception, and MobileNet, was performed. ResNet50 is a convolutional neural network with 50 layers deep [22] with 2.3 × 10^7^ trainable free parameters. VGG16 [35] architecture contains 1.3 × 10^8^ trainable free parameters and 16 trainable layers each of which is composed of filters with spatial size 3 × 3. In this study, the weights of the last five layers were retrained. Inception-v3 [36] is a deep network composed of 48 layers and fewer parameters than VGG16 architecture. This architecture has 2.1 × 10^7^ trainable free parameters. Xception [37] is an extension of the original Inception [38] architecture which replaces the standard Inception’s modules with depth-wise separable convolutions reducing the trainable free parameters to 2 × 10^7^. MobileNet [39] architecture consists of depth-wise and point-wise convolution layers resulting in 3.2 × 10^6^ trainable free parameters.

Accuracy, precision, recall, F1-score, sensitivity, and specificity were used as evaluation metrics for testing the performance of the classifiers. Bellow, we present the mathematical formulations used to calculate the evaluation metrics, where we indicate as TP (true positive) the correct classification of an image as benign, as FP (false positive) the false classification of an image as benign while it is malignant, as TN (true negative) the correct classification of a malignant image and FN (false negative) the false classification of a benign image as malignant [40,41]:

(1) $$accuracy=\frac{TP+TN}{TP+FP+TN+FN}$$

(2) $$precision=\frac{TP}{TP+FP}$$

(3) $$recall=\frac{TP}{TP+FN}$$

(4) $$F1-score=2\times \frac{\left(recall\times precision\right)}{recall+precision}$$

(5) $$sensitivity=\frac{TP}{TP+FN}$$

(6) $$specificity=\frac{TN}{FP+TN}$$

## 3. Results

To evaluate the classification performance of LB-FCN *light* architecture, we adopted the methodology applied in [14] where state-of-the-art convolutional neural networks were employed for solving the three-class classification problem of BS detection on P-Ca patients’ images. These CNNs have already been applied in similar problems of bone metastasis classification in nuclear medicine [10,13,14,15,42,43]. To this end, LB-FCN *light* was compared with ResNet50, VGG16, MobileNet, InceptionV3, Xception, and the fast CNN proposed in [14], namely Papandrianos et al., following 10-fold stratified cross-validation. Table 1 presents the characteristics of the state-of-the-art CNNs that are used in the evaluation. In this procedure, the dataset was partitioned into 10 stratified subsets, from which 9 were used for training and 1 for testing. This was repeated 10 times, each time selecting a different subset for testing until all folds were tested. For training we used the Adam optimizer with a batch size of 32 images, learning rate = 0.001 with first (beta1) and second (beta2) moment estimates exponential decay rate beta1 = 0.9 and beta2 = 0.999. As not all images of the dataset are of the same spatial size, the images were uniformly downsized to 224 × 224 pixels and zero-padded to maintain the original aspect ratio. A minimal data augmentation process was applied, in a form of sample image rotation and rescaling. No further pre-processing step was applied to the input images other than standard pixel normalization between 0 and 1. For the implementation, we used the Keras API from the Python TensorFlow [44] framework. The training was performed on an NVIDIA GeForce GTX 960 GPU equipped with 1024 CUDA cores, 4GB of RAM, and a base clock speed of 1127 MHz.

The comparative classification performance results, which are illustrated in Table 2, Table 3, Table 4 and Table 5, show that the LB-FCN *light* architecture can generalize significantly better compared to conventional pre-trained networks. Specifically, due to its ability to extract multi-scale features, LB-FCN light achieves a 5.8% higher classification performance compared to state-of-the-art [14] network trained exclusively on the same dataset. This is more apparent when compared to malignant (Table 3) and degenerative images (Table 4), where the classes are harder to distinguish compared to healthy class images.

While LB-FCN *light* architecture achieves higher classification results compared to state-of-the-art networks, it is also able to maintain low computational requirements. This is illustrated in Table 5, which includes the computational requirements of all the networks tested in this paper. With respect to the required number of free parameters and FLOPs (see Table 6), LB-FCN *light* computational requirements are significantly lower when compared to the rest of the CNN networks. It should be noted that LB-FCN *light* is more than 10 times lighter compared to MobileNet [39] that is a well-known light-weighted and efficient network especially designed for mobile applications.

## 4. Discussion

In this study, the LBFCN *light* architecture was adopted to identify bone metastasis in the case of patients suffering from prostate cancer based on their whole-body scintigraphy images. The problem was formulated as a three-class classification problem aligned with [14]. The LB-FCN *light* architecture was chosen to address the limitations derived from previous works [9,10,11,12,13,14,15,16] such as:

1. A large annotated dataset of medical images is necessary to achieve strong generalization ability.
2. Abnormalities in images can also be presented due to non-neoplastic diseases. This can lead to low specificity and high sensitivity.
3. The use of deep learning in computer-aided diagnostic systems typically requires significant computational resources, limiting their use to powerful computers.

Due to a lack of publicly available datasets in WBS images of patients with BS, a straightforward comparison with most of the proposed methodologies in the literature remains a challenge. Table 7 summarizes results from the literature review on recent Machine Learning (ML) based BS classification studies. The great majority of the reported approaches employ CNN-based methodologies to implement the classification problem of BS detection. Being a gold standard in BS detection, CNNs have been used in various architectures [10,14,15,42,45] outperforming conventional ML models such as ANNs [11] or LR, DT and SVMs [16]. However, difficulties in comparing their efficacy of the different CNN architectures arise from the fact that each of the aforementioned techniques uses its own dataset that is not publicly available due to privacy reasons. To overcome this barrier, we present a straightforward comparison between the proposed LB-FCN *light* and almost all well-known CNN-based architectures using the same dataset on the BS classification problem. Validation was performed by using a number of evaluation metrics including accuracy, precision, recall, sensitivity, specificity and F1 score indicators.

From the results in Table 2, Table 3, Table 4, Table 5 and Table 6, it arises that the proposed methodology outperforms the previously proposed CNN architectures, as reported in the literature, applied in the specific problem in both classification performance and computational efficiency. Specifically, ResNet50 accomplished a moderate overall accuracy (90.74%) with a low recall for the healthy class (77.7%) while being computationally intensive (with 23.5 × 10^6^ free parameters). VGG16 was the worst performer in terms of computationally efficiency (with 134.2 × 10^6^ parameters to be trained), whereas InceptionV3 gave the lowest overall classification accuracy (88.96%) among the competing CNN algorithms. Xception achieved a relatively high performance (91.54%) with a network of moderate complexity. MobileNet and Gray-based CNN were computationally efficient whereas at the same time they achieved higher overall accuracy compared to the aforementioned CNN approaches. However, MobileNet led to low precision and sensitivity values for the healthy class as well as low recall, F1-Score, and sensitivity in the degenerative class. Moreover, the fast CNN network proposed by Papandrianos et al. resulted in low precision, recall, F1-Score, and sensitivity for the subjects of the degenerative class.

LBFCN light architecture was chosen due to its significantly lower number of free parameters compared to state-of-the-art CNN networks. Furthermore, the results prove that the adopted methodology not only decreases the computational complexity of the model but also increases the accuracy significantly. Compared to the existing methodologies, the following text outlines the main advantageous characteristics of the proposed LB-FCN architecture in light mode. More precisely, LB-FCN *light*:

1. Is capable of generalizing well, even when the availability of training images is limited, due to its multi-scale feature extraction process. This is important in applications where high classification performance is required with limited data. Such applications include computer-aided medical systems, where data availability is limited due to patient privacy legislation.
2. Achieves a high overall classification performance outperforming the state-of-the-art approaches. Specifically, LB-FCN *light* achieved a 97.41% accuracy rate, which indicates that the proposed architecture can detect bone metastasis with almost three times lower error rate (2.59%) compared to the state-of-the-art approach [14].
3. Has a significantly lower number of free parameters (0.3 × 10^6^) and FLOPs (0.6 × 10^6^) compared to conventional approaches enabling its use in embedded and mobile devices, such as tablets and portable diagnostic systems.

The produced results suggest the feasibility of the proposed LB-FCN *light* network to classify bone metastasis using whole-body scans in the field of nuclear medicine. Even though this too effective fully CNN-based network uses a relatively small dataset of patients, this work suggests that bone scintigraphy, incorporating a variety of multiscale feature extraction and a low number of free parameters, can have a considerable effect in the detection of bone metastasis, providing at the same time a potential application in mobile devices.

The main outcome of this study can be summarized as follows: The proposed LB-FCN *light* architecture is powerful enough, in all aspects concerning computational performance, complexity, and generalization, outweighing the CNN architectures previously applied in whole-body image classification problem in bone scintigraphy, as reported in the literature. The validation of the proposed methodology on a small dataset could be considered as a potential limitation of this study since most of the notable accomplishments of deep learning are typically trained and validated on very large amounts of data. Moreover, the insufficiency of the current method to provide explanations on the decisions could also be seen as a limitation since the network is treated as a black box. Future work includes the use of LB-FCN *light* architecture in classifying and localizing possible bone metastasis from bone scans of patients, gathering more images from patients suffering from prostate cancer, as well as patients suffering from other various types of metastatic cancer, such as breast cancer, kidney, and lung cancer.

## 5. Conclusions

A new lightweight deep learning architecture is proposed in this paper for bone metastasis classification in prostate cancer patients. The proposed LBFCN-*light* overcomes the computational burden by using a CNN with a significantly lower number of FLOPs and free parameters. A thorough comparison with several well-known powerful CNNs proved the superiority of the proposed methodology over the current state-of-the-art on identifying bone metastasis. Specifically, LB-FCN *light* was proved at least 6% more accurate and at least 10 times computationally lighter from all the competing algorithms. Overall, the proposed methodology demonstrates a unique potential for enhanced cancer metastasis monitoring and treatment using lighter and at the same time more accurate networks thus facilitating their application on mobile and embedded devices.

## Figures and Tables

**Table 1:** Characteristics of the networks used in the evaluation.

Network	Characteristics
ResNet50 [22]	pixel size (250×250×3), batch size = 64, dropout = 0.2, global average pooling, dense nodes 1024 × 1024, epochs = 200
VGG16 [35]	pixel size (250×250×3), batch size = 32, dropout = 0.2, flatten, dense nodes 512 × 512, epochs = 200
MobileNet [39]	pixel size (300×300×3), batch size = 32, dropout = 0.5, global average pooling, epochs = 200
InceptionV3 [36]	pixel size (250×250×3), batch size = 32, dropout = 0.7, global average pooling, dense nodes = 1500×1500, epochs = 200
Xception [37]	pixel size (300×300×3), batch size = 16, dropout = 0.2, flatten, dense nodes = 512×512, epochs = 200
Papadrianos et al. [14]	pixel size (400×400×3), batch size = 32, dropout = 0.2, 16–32–64–128 dense nodes = 32, 16, epochs = 300
LB-FCN *light* [33]	pixel size (224×224×3), batch size = 32, global average pooling, epochs = 200

**Table 2:** Comparative classification performance for the healthy class.

Network	Precision	Recall	F1-Score	Sensitivity	Specificity
ResNet50 [22]	0.994	0.777	0.866	0.825	0.997
VGG16 [35]	0.952	0.844	0.896	0.855	0.988
MobileNet [39]	0.890	0.990	0.936	0.857	0.960
InceptionV3 [36]	0.884	0.958	0.916	0.959	0.947
Xception [37]	0.958	0.908	0.931	0.913	0.988
Papadrianos et al. [14]	0.950	0.938	0.942	0.938	0.978
LB-FCN *light* [33]	0.972	0.978	0.975	0.978	0.992

**Table 3:** Comparative classification performance for the malignant disease class.

Network	Precision	Recall	F1-Score	Sensitivity	Specificity
ResNet50 [22]	0.904	0.972	0.934	0.971	0.921
VGG16 [35]	0.952	0.950	0.952	0.949	0.960
MobileNet [39]	0.946	0.941	0.944	0.940	0.952
InceptionV3 [36]	0.902	0.922	0.911	0.920	0.909
Xception [37]	0.964	0.932	0.946	0.937	0.909
Papadrianos et al. [14]	0.948	0.928	0.938	0.927	0.960
LB-FCN *light* [33]	0.979	0.979	0.979	0.978	0.984

**Table 4:** Comparative classification performance for the degenerative class.

Network	Precision	Recall	F1-Score	Sensitivity	Specificity
ResNet50 [22]	0.830	0.882	0.846	0.881	0.902
VGG16 [35]	0.836	0.914	0.872	0.913	0.917
MobileNet [39]	0.938	0.856	0.888	0.857	0.960
InceptionV3 [36]	0.848	0.754	0.786	0.755	0.937
Xception [37]	0.820	0.936	0.904	0.934	0.925
Papadrianos et al. [14]	0.862	0.894	0.874	0.894	0.933
LB-FCN *light* [33]	0.970	0.967	0.968	0.967	0.984

**Table 5:** Overall classification accuracy comparative results.

	ResNet50 [22]	VGG16 [35]	MobileNet [39]	InceptionV3 [36]	Xception [37]	Papandrianos et al. [14]	LB-FCN *light* [33]
Accuracy	90.74%	90.83%	91.02%	88.96%	91.54%	91.61%	**97.41%**

**Table 6:** Computational performance comparison.

	FLOPs (×10^6^)	Trainable Free Parameters (×10^6^)
ResNet50 [22]	47.0	23.5
VGG16 [35]	268.5	134.2
MobileNet [39]	6.4	3.2
InceptionV3 [36]	43.5	21.8
Xception [37]	41.6	20.8
Papadrianos et al. [14]	13.1	6.5
LB-FCN *light* [33]	**0.6**	**0.3**

**Table 7:** Summarized results of state-of-the-art ML approaches for bone metastasis (BS) classification.

Studies	Year	ML Method	Classification Problem	Results
[10]	2020	Deep CNNs	2 classes: absence or presence of bone metastasis	accuracy of 89.00%, F1-score of 0.893, and Sensitivity of 92.00%
[14]	2020	CNN	2 classes: BS metastasis in prostate patient or not 3 classes: (a) benign, (b) malignant and (c) degenerative	overall classification accuracy 91.61% ± 2.46% accuracy regarding normal, malignant and degenerative changes: 91.3%, 94.7% and 88.6%
[15]	2020	CNN	2 classes: BS metastasis in prostate patient or not	97.38% classification testing accuracy and 95.8% average sensitivity
[9]	2019	Parallelepiped algorithm	2 classes: absence or presence of bone metastasis	87.58 ± 2.25% classification accuracy and 0.8367 ± 0.0252 *κ* coefficient
[12]	2019	Modified Fully CNN	Segmentation of the BS area	69.2% intersection over union rate and 79.8% true positive rate
[13]	2019	CNN	2 classes: metastasis of breast cancer or not	classification accuracy of 92.50%, 95% sensitivity
[11]	2016	CADBOSS (ANNs)	2 classes: absence or presence of bone metastasis	92.30% accuracy, 94% sensitivity and 86.67% specificity
[16]	2016	LR, DT and SVM	2 classes: absence or presence of bone metastasis	LR, DT, and SVM classification accuracy was 79.2%, 85.8% and 88.2%
